# Student Self-perception on Digital Literacy in STEM Blended Learning Environments

**DOI:** 10.1007/s10956-022-09956-1

**Published:** 2022-02-02

**Authors:** Branda Le, Gwendolyn A. Lawrie, Jack T. H. Wang

**Affiliations:** School of Chemistry and Molecular Bioscience, University of Queensland (UQ), SCMB, Building 68 Cooper Road, Brisbane, QLD 4067 Australia

**Keywords:** Post-secondary education, Distance education and online learning, Blended teaching/learning strategies, Data science applications in education, Digital literacy

## Abstract

As students transition into tertiary blended learning environments, their digital literacy in terms of technical capabilities have potential to impact on their access to digital resources. The first foundational year of STEM degrees includes compulsory courses across a broad range of scientific areas, each of which incorporates online technology in a discipline-specific manner. Given the diversity of online resources that STEM students need to access across their first-year coursework, this study applies learning analytical methods to determine whether students’ perceived level of digital literacy has an effect on their navigation of learning management systems (LMS) and overall academic performance. The frequency and nature of LMS interactivity were examined across four first-year STEM courses offered in the same semester at a single institution, using a K-means cluster analysis to group student responses. It was observed that high achieving students accessed LMS resources more frequently than mid or low-achieving students across all four STEM courses. Students’ perceived level of digital literacy was collected via survey (*n* = 282), and students were sorted high (*n* = 106) and low-level (*n* = 176) of perceived digital literacy—HDL and LDL, respectively. HDL students were not consistently found in the high-achieving academic group and did not perform better in their overall grade when compared to LDL students. LDL students were observed to perform better in specific online assessment tasks, which may be attributed to their increased frequency of LMS interactivity. These findings highlight the delicate balance between students’ perceived level of digital literacy, motivation for engaging with online learning environments, and academic performance.

## Introduction

An immediate challenge for the twenty-first century is the integration of technologies in online and blended learning (BL) strategies to underpin contemporary pedagogies and teaching practices. Learning environments in higher education are now reliant on their adaptation into a digital world, and therefore need to utilise the full potential of learning technologies (Cook & Thompson, 2014; Garrison & Kanuka, 2004). This process had evolved slowly in the structure of academic courses (Bernard et al., 2014) prior to the precipitous global shift into online learning environments across all education sectors that was catalysed by the response to the COVID-19 pandemic in 2020. A pivot into emergency response teaching (ERT) occurred as teachers responded in practice (Bozkurt & Sharma, 2020; Bozkurt et al., 2020), including at our own institution (Slade et al., 2021), emphasising the necessity to better understand the ways that students engage with online learning in relation to their academic outcomes. The transition from secondary to tertiary learning contexts represents a key focal point for student engagement in terms of exploring whether additional scaffolding is required to better support students as they become independent learners in digital learning environments. This current study, completed immediately prior to the pandemic, aimed to inform instructional design through an in-depth analysis of factors that contribute to patterns of student interactivity with BL resources and learning outcomes in first semester, first year courses. Student perceptions and interactivity are considered through comparison of four STEM courses that share a common learning management system (LMS). While these courses contained a wide variety of online learning resources, it has been previously noted that BL strategies in general have a positive impact on academic achievement in STEM courses (Vo et al., 2017).

### Blended Learning in STEM courses

The shift from traditional, didactic, teacher-centred classrooms towards active, student-centred learning environments had advanced through enhanced online and digital learning technologies and resources (Bonk & Graham, 2012; Castro, 2019). Prior to the pandemic ERT, BL as a paradigm had been evolving into the new ‘traditional’ teaching model (Brown, 2016). While there were many affordances of BL environments, evidence had indicated that students value face-to-face interactions the most (Akkoyunlu & Soylu, 2006). Students expressed low enthusiasm regarding technology-based learning based on a perception of increased workloads (Tune et al., 2013).

Science, technology, engineering, and mathematics (STEM) fields are widely regarded as vital to a national economy, yet STEM-related courses continue to face challenges in terms of attraction, persistence, and retention of students (Chen, 2013; Sithole et al., 2017). There is a large body of literature reporting outcomes from a range of blended learning interventions that is beyond the scope of a review here; however, we can provide several recent examples. The BL experience has been observed to have a significantly higher positive effect on academic achievement in STEM courses compared to non-STEM courses (Vo et al., 2017). Comparison between different types of resources and activities have identified positives for example in terms of pre-class content delivery and virtual laboratory learning (Hu-Au & Okita, 2021). Prior to the pandemic, a systematic review of virtual laboratories in science and engineering courses revealed that positive outcomes were often linked to novelty rather than pedagogical design improving student motivation (Reeves & Crippen, 2021). The emphasis on practical-based classes in STEM disciplines can increase the complexity of BL implementation (Bernard et al., 2014), which can be further compounded by the variability in the effectiveness of teaching innovations across different STEM disciplines (Ruiz-Primo et al., 2011). Recently, a comparison of face-to-face, blended, flipped, or online environments in a physiology course has indicated that flipped classrooms had improved student perceptions of flexibility and self-efficacy but negative perceptions could cancel these outcomes (Thai et al., 2020). The effectiveness of BL still remains inconclusive; however, the body of evidence is growing to demonstrate positive outcomes for this approach representing the ‘new normal’ post-COVID-19 (Ma & Lee, 2021).

Enrolment in a first-year STEM discipline course has potential to introduce a risk factor, with the inclusion of learning technologies in a BL environment influencing retention or attrition (Shelton et al., 2017). The online first-year experience has received recommendations for best practice in orientation and supporting students with the aim of improving retention and academic success (Korstange et al., 2020). While academic outcomes are important, understanding non-academic outcomes in BL environments such as students’ self-regulated learning strategies, motivation, cognitive engagement, and resource management strategies is important (Anthonysamy et al., 2020).

### The Role of Digital Literacy in STEM BL

Digital literacy as an attribute goes beyond simply searching for, and identifying, digital information; it combines the ability to assimilate and understand information from various digital sources (Ng, 2012; Tang & Chaw, 2016). Digital access, content creation, and resource sharing are online tasks that today’s students are generally familiar with from prior experience in learning and social media (Tang & Chaw, 2016). However, to be digitally literate, one needs to not only to be able to use technology on a social level, but also be able to scrutinise and integrate digital information. This is where the issue lies for today’s students who are often incorrectly referred to as ‘digital natives’ based on their assumed technological skills (Margaryan et al., 2011). However, what is seen is that while students are able to display expertise in conventional technology tools, they are unable to effectively assemble and comprehend information for learning purposes (Tang & Chaw, 2016). Familiarity with technology alone is not enough for success in learning, having the right competencies and attitudes is equally important (Margaryan et al., 2011). Moreover, digital literacy is known to significantly contribute to student self-efficacy (SE) skills; these greatly support the requirement of self-directed learning in a BL experience (Prior et al., 2016). In STEM disciplines, self-regulated learning, epistemic cognition, and digital literacy have been found to relate to learning (Greene et al., 2018) and underpin students’ information organising skills (Demirbag & Bahcivan, 2021). A recent study comparing psychology and veterinary science undergraduate students’ self-reported digital capabilities during COVID-19 lockdown found that students with a high level of self-regulation and digital capabilities were able to remain focussed and engaged (Limniou et al., 2021). No difference was found between the academic performance on comparison of the two disciplines.

### Student Interactions with Content in BL

Learning management systems (LMS) such as Blackboard^™^ Learn, Moodle^™^, WebCT, and Sakai complemented by virtual learning environments (VLE) such as EdX^®^ Edge and Coursera have become the central hubs through which digital resources and tools are accessed by students. The terms LMS and VLE are often used interchangeably in literature; for example, Moodle is cited as both a LMS (Cohen, 2017; Kadoić & Oreški, 2018; Kotsiantis et al., 2013) and a VLE (Mogus et al., 2012). Pinner (2011) suggests that there is a functional distinction between the two—LMS’s aim is to distribute information to users, resulting in a mainly one-sided interaction, whereas VLE allow users to interact with content creators, educators, and instructors as well through a range of activities (Pinner, 2011; Weller, 2007).

The analysis of student interactions with content in digital environments such as LMS platforms involves the collection of clickstream data. Early studies focused on measuring activity related to a singular action (Davies & Graff, 2005), but the number of monitored activities has increased along with the sophistication of the analyses. Certain interactions have the potential to impact student performance more than others, but there is mixed evidence in the literature to pinpoint which indicators are predictive of high versus low achieving students.

Mogus et al. (2012) observed a variety of student interactions: assignment/course view, assignment upload time, posts to, and views of forums for two separate cohorts of undergraduate education students. The authors demonstrated that students with a higher number of interactions logged in the LMS (Moodle) achieved higher final marks (Mogus et al., 2012). In separate studies, at-risk students were observed to engage in reduced online activity relative to the rest of the student cohort (Cohen, 2017; Kadoić & Oreški, 2018; Kotsiantis et al., 2013).

The variables of assignment view, course content view, and forum view have been found to be interactions that correlated with academic performance (Mogus et al., 2012). This finding is, to an extent, supported by the Kotsiantis et al. (2013) who measured the engagement of three cohorts of information and communication technologies’ (ICT) undergraduates and observed that higher assignment, forum, and course content views were linked to excellent grades (Kotsiantis et al., 2013). Students who viewed their user profile, which featured information of their overall progress as feedback, also received higher grades.

Cohen (2017) aimed to identify at-risk students based on their online activity, and speculated that course characteristics (e.g. elective or compulsory) were a contributing factor in their results. Soffer and Nachmias (2018) hypothesised that unless the enrolments were randomised, students who have a technological orientation may be selecting courses (face-to-face or online) where they could use their background as an advantage (Soffer & Nachmias, 2018). These effects may not be evident in a BL course, as students who struggle online have an opportunity to compensate through face-to-face time and are not selecting courses based on requirements for technological skills. When corroborating interaction data with student perceptions, both Mogus et al. (2012) and Kotsiantis et al. (2013) found that course failure was associated with negative attitudes towards VLE.

Student attrition can be most accurately predicted using interaction data leading up to the last two weeks of semester (Shelton et al., 2017); however, this is often too late for successful intervention. Evidence also suggests that the magnitude and type of interaction observed in a BL course is lower than for a completely online course and cannot establish a relationship to academic performance (Agudo-Peregrina et al., 2014). We aim to investigate whether valuable insights can still be gained into student behaviour and outcomes by exploring multiple STEM courses in parallel.

### Study Aims

The current study aims to apply learning analytical methods to determine whether students’ perceived level of digital literacy has an effect on their academic performance by examining the frequency and type of student interactions with the LMS across four first-year STEM courses offered in the same semester at a large tertiary institution in Australia. Weekly variations in student interactivity with content, specifically changes in interactivity near assessment deadlines, were investigated. Evidence that digital literacy can impact on students’ approaches to learning (Greene et al., 2018) and self-reported engagement can relate to academic success (Soffer & Nachmias, 2018) indicated that adoption of k-cluster analysis to explore the existence of distinct groups in each variable was merited. We explore the following research questions in this study:

- Research question 1: what is the nature of the relationship between student interactivity and academic performance when comparing blended STEM courses across four disciplines?
- Research question 2: to what extent does students’ perceptions of their digital literacy relate to their level of interaction with digital learning environments in blended learning STEM courses?

## Methods

The research tools utilised in this study triangulate students’ perceptions of digital literacy, their academic performance in foundational first year STEM courses, and clickstream learning analytics data from these online learning environments. These data sources collectively address both research questions 1 and 2 and are outlined below.

### Research Participants

The participants were 234 students enrolled in at least one out of four first-year STEM courses offered at the same institution between February and July in 2019. The courses were delivered for 17 weeks and included a one-week mid-semester break after week 8, and three examination period weeks at the end of the semester. The courses included a biology course (*n* = 128), a chemistry course (*n* = 121), a mathematics course (*n* = 61), and a quantitative science course (*n* = 117), all of which serve as foundational first year prerequisites for a bachelor’s degree in science at a research-intensive university. Given the foundational nature of these four courses within the same degree programme, students are often concurrently enrolled in more than one of these courses in their first semester of university study (Table 1). Ethical approval for all procedures was granted by the institution ethics committee (Project # 2,016,001,757), and informed consent was obtained from participants.

**Table 1:** Descriptive statistics of participants

**Category**	**Sub-category**	**Frequency**	**Percentage**
Age	≤ 17	90	38.46%
	18–19	116	49.57%
	20–21	14	5.98%
	22 ≤	14	5.98%
Number of participating Courses enrolled	Only 1 course	110	47.01%
	2 courses	57	24.36%
	3 courses	65	27.78%
	4 courses	2	0.85%
Number of university semesters completed	≤ 1	200	85.47%
	2–3	20	8.55%
	4–5	8	3.42%
	6–7	3	1.28%
	8 ≤	3	1.28%
Field of study	Biological sciences	71	30.34%
	Health sciences	31	13.25%
	Mathematics	28	11.97%
	Science	47	20.09%
	Other/dual programme	57	24.36%

### Data Collection

#### Survey Data

Every student enrolled across each of the four courses was invited to complete an online survey at the beginning of the semester through an email sent with permission of the course coordinator (students who were enrolled in multiple courses only received the invitation once). Students gave informed consent for their de-identified academic performance data to be included in this study, and their perceived level of digital literacy was measured using an 8-item Likert-type scale survey (Ng, 2012).

#### Academic Performance

Academic performance for consenting students was collected from university databases and/or from course coordinators with their permission. To formulate academic performance groups, students were aggregated based on their final grade in each course (measured on a 1–7 scale, with 7 being the highest and results < 4 equating to a fail). High-achieving students received a 6–7, mid-achieving students received a 5–4, and low-achieving students received < 4. Similar student groupings have been observed in previous studies (Davies & Graff, 2005; Kotsiantis et al., 2013; Tune et al., 2013), as this accounts for a finer level of granularity across multiple tiers of academic performance beyond simply pass or fail. High-, mid-, and low-achieving student groups are independently formulated for each course.

#### Learning Analytics Clickstream Data

Learning analytics is the measurement, collection, analysis, and reporting of data about learners and their contexts with the purpose of enhancing learning environments (Ferguson, 2012; Tang & Chaw, 2016). Temporal learning analytics data are proving to be highly useful in revealing insights into students’ application of learning strategies, including the role of assessment, and self-regulated learning skills in terms of task orientation and cognitive strategies (Fan et al., 2021). Student engagement with LMS or VLE produce clickstream data that enables exploration of the student-content interaction which is an important aspect of BL (Bernard et al., 2014). It is feasible to extract a vast quantity of interactions in the form of personal, systems, and academic data regularly from the LMS. Institutions are then faced with the issue of how to aggregate this ‘big data’ in a significant manner (Ferguson, 2012) and provide scaffolding for student learning.

In this study, Blackboard clickstream data, including 786,583 clicks, was collected, aggregated, and parsed using R. Clicks were grouped based on features including timestamp, student, and course, then sorted into academic weeks. The clicks were then grouped based on interactivity measures summarised in Table 2, which have been previously correlated to academic performance (Kotsiantis et al., 2013). Clicks that corresponded to navigational prompts (e.g. ‘Click OK to confirm’) were not included in any of these interactivity measures, but are included in the analysis under ‘Total Interactions’ for each course.

**Table 2:** The interactivity measures analysed in the current study

**Interactivity measure**	**Description**
Assignment view	Clicks related to viewing/submitting assessment
Course content view	Clicks related to viewing general course content
Peer interaction	Clicks related to viewing or posting in forums
User view	Clicks related to viewing student progress or profile
Total interaction	The total number of interactions for each course, including navigational prompts

### Data Analysis

#### Survey Data

To identify students’ level of digital literacy and formulate digital literacy groups, *k*-means cluster analysis was applied to items in the ‘Digital Literacy’ scale. The optimum number of clusters was identified by plotting a scree plot of the sum of squares and identifying the point at which the marginal change decreases: the ‘elbow’ (Jackson, 1993).

#### Clickstream and Academic Performance Data

A Mann–Whitney *U* test was conducted to compare the digital literacy groups for both variables and identify significant relationships to examine the effect of digital literacy on interactivity and academic performance. Prior analysis of academic performance data, paired sample *t*-test was conducted on the overall grade of students enrolled in more than one course to compare their performance across the courses they were enrolled in (Field, 2013). A Kruskal–Wallis test was applied to the interactivity measures of the high-, mid-, and low-achieving groups to determine the relationship between academic performance and interactivity; a non-parametric Dunn’s multiple comparisons test, the non-parametric analogue of a *t*-test, was also conducted to identify which groups were significantly different for each significant interactivity measure. The significance before and after the Bonferroni correction was reported (Dinno, 2015; Dunn, 1961), along with the mean, standard error of mean (± SEM), and the eta-squared (η^2^) non-parametric measure of effect size. Benchmarks for estimating small (η^2^ = 0.01), medium (η^2^ = 0.06), and large (η^2^ = 0.14) effect sizes have been previously described (Cohen, 1988; Lakens, 2013). To observe whether any association between digital literacy, academic performance, and interactivity is present, a non-parametric Spearman’s correlation test was conducted (Villagrá-Arnedo et al., 2017).

## Results

### Research Setting

**Fig. 1:**
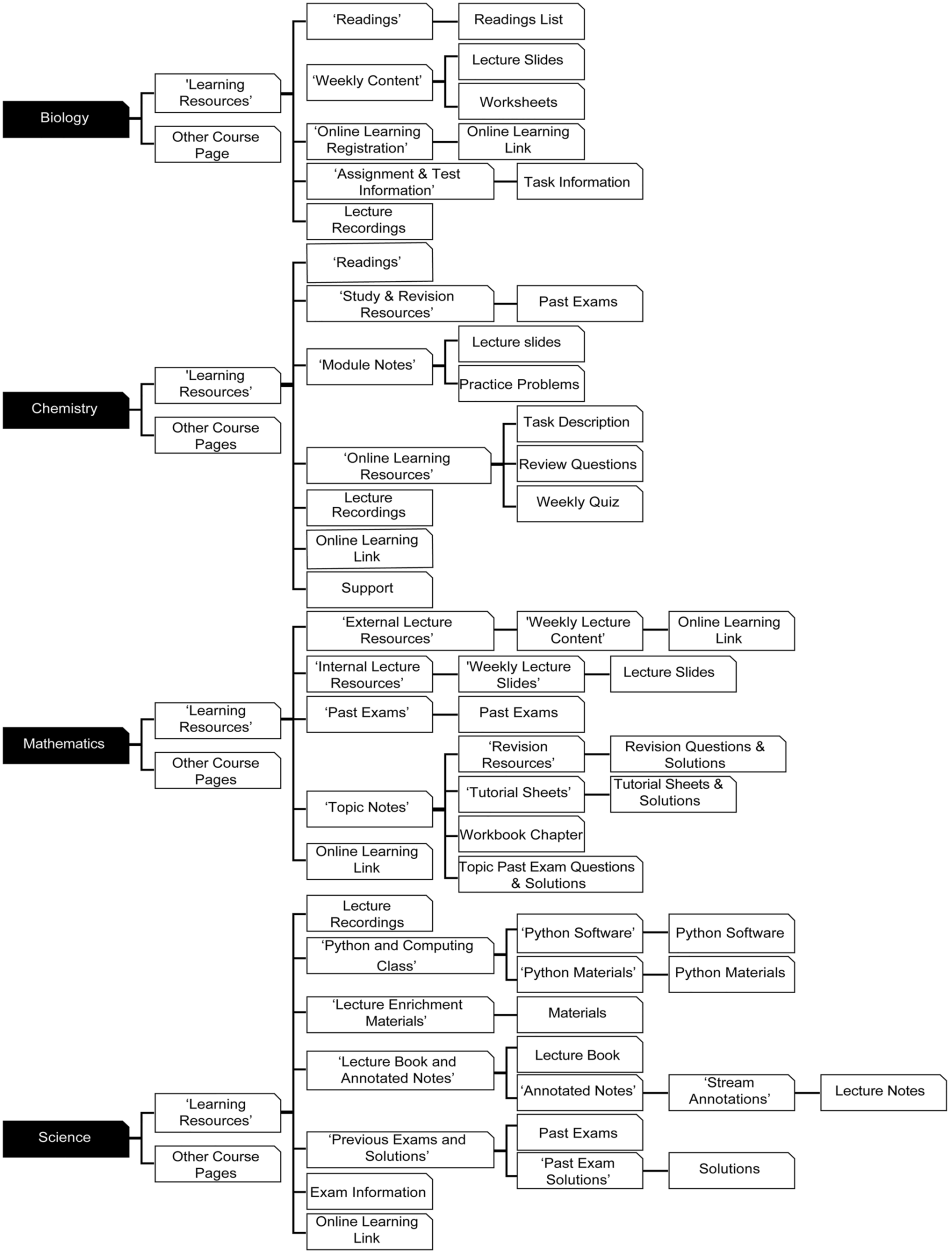
Courses’ ‘Learning Resource’ page navigation on Blackboard Learn

To explore student interactivity and academic performance across STEM blended courses, this study examined STEM courses offered at the same institution involving four disciplines—biology, chemistry, mathematics, and quantitative science. All four courses use Blackboard^™^ as the virtual learning environment linking to other digital platforms through learning tool integration. To evaluate each course delivery mode, the courses’ learning activities were collated from their electronic course profiles and further clarified by each respective course coordinator. All four courses were delivered in a blended mode, including both online and face-to-face learning activities. There was minimal variation in the face-to-face components of the courses, comprising of 1–3 lectures and 2–3 additional contact hours per week, which included tutorials, laboratory practical sessions, and coding workshops. The online resources provided in the four courses spanned across learning materials, practical worksheets, assessment, and supplementary learning resources, which were hosted through Blackboard and incorporated into different face-to-face learning activities for each course (Table 3). A summary of how each course’s content navigation is structured through Blackboard is outlined below (Fig. 1). While each course contained a range of different pages, the ‘Learning Resources’ page has been highlighted as the majority of student-content interactions occurred through this area. The online resources for biology and chemistry were contained within 3 levels of navigation, whereas mathematics and science had 4 and 5 levels of navigation, respectively.

**Table 3:** Range of online resources offered in the participating courses

**Resources**	**Sub-category**	**Biology**	**Chemistry**	**Mathematics**	**Science**
Lecture/Learning	Lecture recordings	✔	✔	✔	✔
	Lecture notes	✔	✔	✔	✔
	Textbook	✔	✔	✔	
	Past exams		✔	✔	✔
	Simulations		✔	✔	
	Programming help				✔
Practical	Practical manual	✔	✔		
	Tutorial sheets	✔			✔
	Workbook			✔	✔
Assessment	Online modules		✔	✔	✔
	Course hurdles	✔	✔	✔	✔
	Online quizzes		✔	✔	✔
Supplementary	Supplementary videos	✔	✔	✔	
	Peer-assisted study		✔		✔
	Blackboard forums	✔	✔	✔	✔
	External forums	✔	✔	✔	✔

To obtain a holistic overview of student interactivity with the online resources offered across each course, Blackboard clickstream data was accessed to determine the total interactions per student across the whole semester. Chemistry, mathematics, and science all exhibited greater than 2000 total interactions per student, whereas biology had fewer than 1000 total interactions per student throughout the semester. Similar trends were observed when the interactivity data is filtered for students concurrently enrolled in multiple courses, with the most common three course combination being biology, chemistry, and science (62 out of 234 students). For this pool of students, the total interactions for biology (886.08 ± 53.73) remained lower than that of chemistry (1920.08 ± 129.148) and science (1939.79 ± 103.56). This may be partially explained by the simpler navigational complexity of the online learning environment presented in Blackboard for biology (Fig. 1), as well as the absence of past exams, simulations or programming assistance, and online quizzes delivered through its LMS. In contrast, these online resources are found in the LMS for the other three courses (Table 3). This disparity in student interactivity and its potential connection to course-based assessment warranted further investigation.

### Research Question 1—Academic Performance and Interactivity

**Fig. 2:**
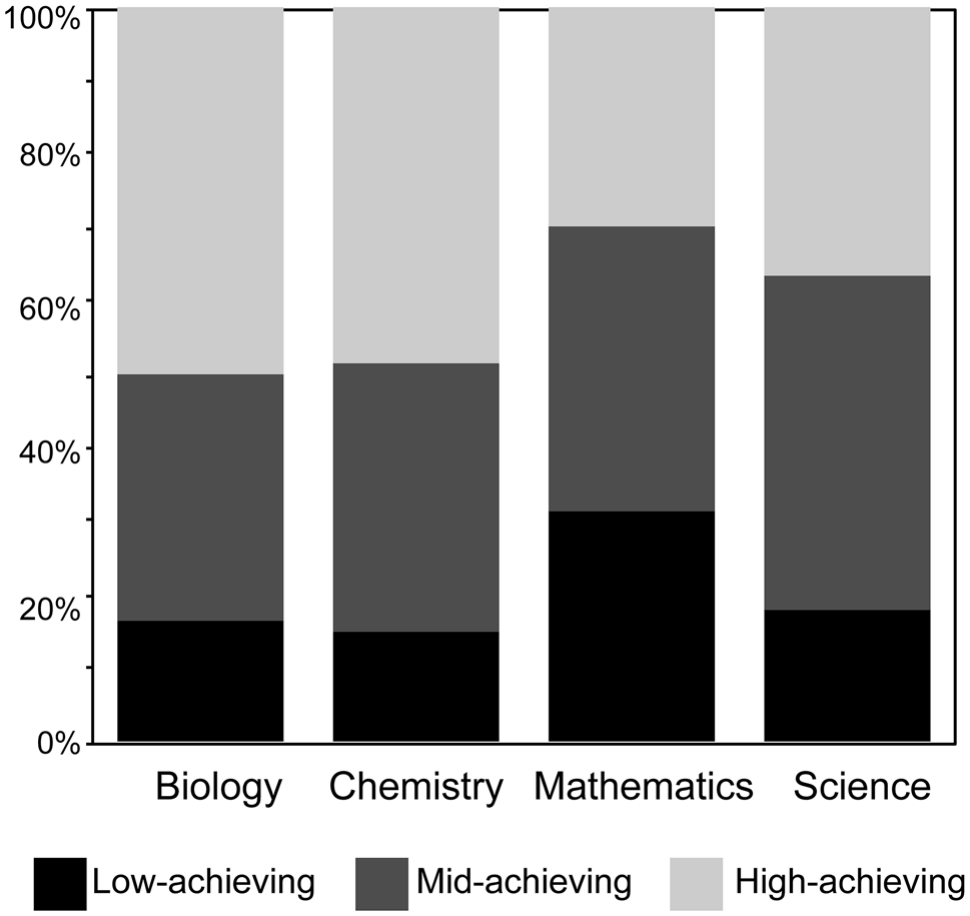
Distribution of high-, mid-, and low-achieving students (*n* = 234)

Given the positive correlation between academic performance and interactivity as identified by previous studies, we chose to further examine this relationship within the context of our study. Students were grouped in high-, mid-, and low-achieving tiers for each course according to their course-specific grades, and the distribution in academic performance is displayed in Fig. 2. To account for variability in course difficulty, we examined the academic performance of students enrolled in more than one of the participating courses within the study, which represented 53% of the participant pool. Students enrolled in biology, chemistry, and science (*n* = 62) on average had an overall score of 68.23 ± 2.95%, 70.15 ± 2.91%, and 65.68 ± 2.81%, respectively, all of which indicated a mid-achieving student. Similar trends in performance were observed for students enrolled in different 2-course combinations; the only disparity was observed in students enrolled in both chemistry (78.97 ± 3%) and mathematics (64.05 ± 2.85%), but given the small group size (*n* = 5), this trend was inconclusive. While this does not account for students’ perceptions of each course’s difficulty during the semester, the similar performance outcomes at the end of the semester for students enrolled in more than one of the participating courses provide this study with a baseline for comparison.

The type and frequency of assessment and how it is delivered in relation to the LMS varied across the 4 courses. The ‘In-Class Quiz’ for the biology course is not completed through the LMS but instead on paper during face-to-face class time, which again may have contributed to the lower overall clicks compared to the other 3 courses. For the chemistry course, the ‘BL Tasks’ are online activities that could include digital whiteboard (Padlet) submissions and participation in online group chats explaining concepts. Additional assessment in the chemistry course included ‘Weekly Online Quizzes’ and fortnightly ‘Lab Reports’ which were digital worksheets/reports based on the ‘Practical Participation’ as well. Similarly, in the mathematics course, there were weekly ‘Computer Exercises’ that were required to be completed prior to ‘Practical Participation’. Finally, also in the mathematics course, during weeks 4, 6, and 10, multiple assessment items were due. All courses featured summative ‘Exams’ which were traditional paper-based end-of-semester exams conducted under on-campus invigilated conditions without requiring access to the LMS.

**Fig. 3:**
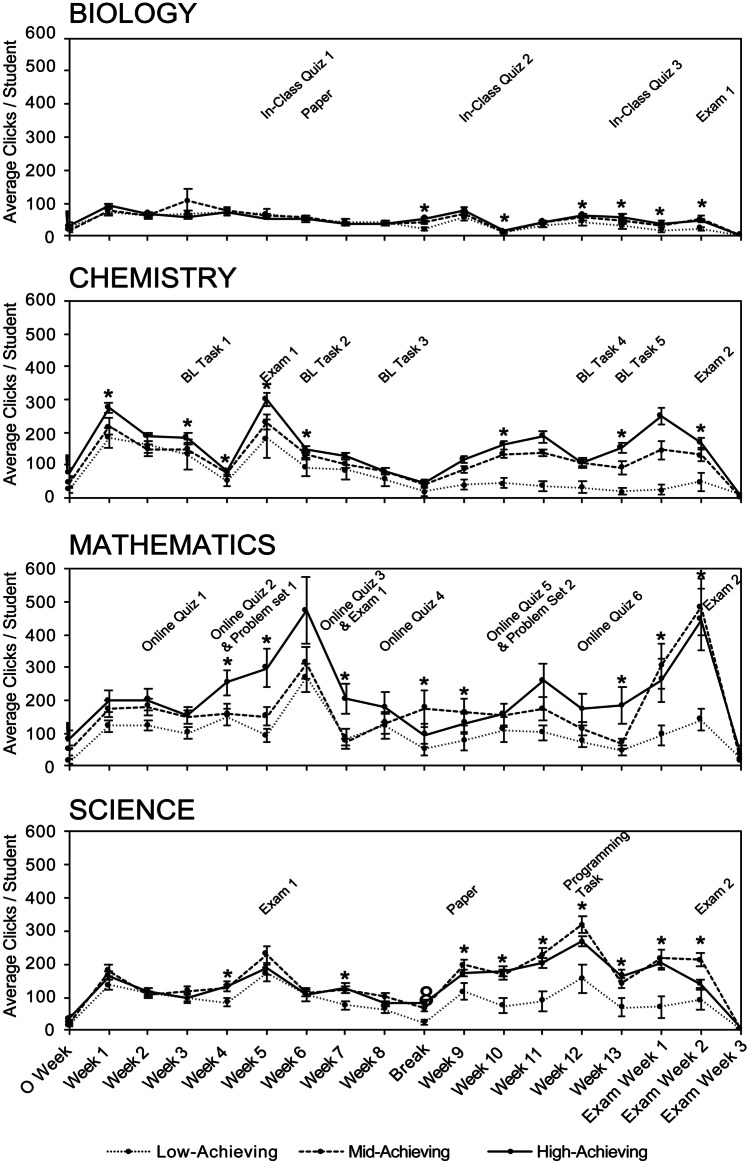
Comparison of total clicks across the semester for high-, mid-, and low-achieving students. Average total clicks for high-, mid-, and low-achieving student ± SEM for each week of the semester. **A** Biology (*n* = 128). **B** Chemistry (*n* = 121). **C** Mathematics (*n* = 61). **D** Science (*n* = 117)

Student interactivity across the semester was visualised through mean weekly ‘Total Interaction’ (Fig. 3) and a Kruskal–Wallis test was conducted on each student’s total weekly clicks to identify potential differences between academic performance groups. High-achieving students were observed to interact more with the LMS than other students in weeks corresponding to assessment deadlines as well as weeks without scheduled assessment. Across all 4 courses, low-achieving students consistently exhibited the lowest weekly LMS interactivity out of all the academic performance groups, with the biggest disparity between low- and high-achieving students being observed in chemistry and mathematics (Fig. 3). This may be attributed to the higher number of summative assessment items in chemistry and mathematics (8 and 10 assessment tasks, respectively, compared to 5 for biology and 4 for science) and the weeks in which statistically significant differences are observed between academic performance groups are evenly spread out throughout the semester. In contrast, the weeks in which statistically significant differences were observed between high-, mid-, and low-achieving students in biology and science are more concentrated towards the second half of the semester. The lower overall interactivity in biology irrespective of student performance groups may reflect the difference in its LMS structure and the relative absence of interactive online resources compared to the other 3 courses (Table 3). Collectively, these results suggest that the frequency of assessment deadlines may be a factor in student interactivity, and the corresponding impact on student online engagement will ultimately influence academic performance.

Interactivity measures for each academic performance group were also observed across the whole semester (Table 4). A Dunn’s multiple comparison post hoc test was completed to identify the differences between each group (Table 5). In the biology course, high-achieving students averaged more clicks than low-achieving students in ‘Assignment View’, a statistically significant difference with a medium effect size (η^2^ = 0.1119). In chemistry, consistent and significant differences were observed across all three performance groups. High-achieving students scored higher than low-achieving students in all interactivity measures (assignment view, course content view, peer interactions, user view) with a medium to large effect sizes for each comparison. In relation to their total online interactions within the course, high-achieving students averaged more total clicks than both mid and low-achieving students (η^2^ = 0.1193 and η^2^ = 0.254, respectively), and mid-achieving students averaged more than low-achieving students albeit with a small effect size (η^2^ = 0.0334) (Table 5).

**Table 4:** Descriptive statistics and non-parametric Kruskal–Wallis statistical analysis of student academic performance groups

**Course**	**Interactivity measure**	**Low mean ± SEM**	**Mid mean ± SEM**	**High mean ± SEM**	**Sig**
Biology	Assignment view	52.3 ± 6.8	71 ± 5.4	73 ± 3.4	0.015*
	Course content view	399.6 ± 65.9	525.3 ± 53.6	497.6 ± 26.5	0.077
	Peer interaction	7.4 ± 3	2.5 ± 1	5.9 ± 1.9	0.457
	User view	43.4 ± 8.1	50 ± 3.7	59.7 ± 6.8	0.334
	Total interaction	694.3 ± 98.7	898 ± 71.5	912.2 ± 46.6	0.053
Chemistry	Assignment view	89.6 ± 16.7	128.4 ± 6.7	131.6 ± 5	0.006*
	Course content view	371.1 ± 71.3	620.7 ± 56.2	705.7 ± 41.1	0.000*
	Peer interaction	19.2 ± 5.7	40.7 ± 7.3	43.4 ± 6.3	0.009*
	User view	36.5 ± 8.2	62.2 ± 4.6	70.4 ± 5.1	0.002*
	Total interaction	1370.2 ± 257.9	2150.7 ± 154.5	2544.6 ± 118.5	0.000*
Mathematics	Assignment view	97.3 ± 16.7	102 ± 13.2	78.3 ± 9.9	0.695
	Course content view	1133.4 ± 168.7	1831.4 ± 201	2275.5 ± 328	0.007*
	Peer interaction	2 ± 1.1	18 ± 7.6	13 ± 5.1	0.262
	User view	70.1 ± 12.7	179.6 ± 34.5	196.4 ± 36.4	0.003*
	Total interaction	1790.3 ± 240.2	3027.7 ± 328.7	3782.1 ± 555.1	0.003*
Science	Assignment view	129.6 ± 16.3	209.2 ± 9.9	200.5 ± 9.9	0.000*
	Course content view	560.1 ± 73.8	890.8 ± 76	749.2 ± 39.5	0.009*
	Peer interaction	17.6 ± 7.3	17.3 ± 4.8	25.1 ± 9.5	0.077
	User view	50 ± 9.2	72.8 ± 5.7	81.2 ± 6.5	0.004*
	Total interaction	1641.8 ± 235	2682.3 ± 157.3	2438.8 ± 116.3	0.001*

**Table 5:** Dunn’s multiple comparison between academic performance groups (*n* = 234)

**Course**	**Interactivity measure**	**Academic performance level**	**Comparison**	**Sig**	**Adjusted Sig**	**η**^**2**^
Biology	Assignment view	High High	Mid	0.369	1.000	0.0737
			Low	0.004*	0.011*	**0.1119**
		Mid	Low	0.038*	0.113	0.0008
Chemistry	Assignment view	High High	Mid	0.012*	0.037*	**0.0979**
			Low	0.001*	0.004*	**0.1628**
		Mid	Low	0.400	1.000	0.0008
	Course content view	High High	Mid	0.094	0.282	0.119
			Low	0.000*	0.000*	**0.2264**
		Mid	Low	0.005*	0.016*	**0.0154**
	Peer interactions	High High	Mid	0.346	1.000	0.0732
			Low	0.002*	0.007*	**0.0916**
		Mid	Low	0.346	1.000	0.0013
	User view	High High	Mid	0.310	0.929	0.135
			Low	0.000*	0.001*	**0.1737**
		Mid	Low	0.001*	0.025*	**0.0158**
	Total interaction	High High	Mid	0.012*	0.036*	**0.1193**
			Low	0.000*	0.000*	**0.254**
		Mid	Low	0.012*	0.037*	**0.0334**
Mathematics	Course content view	High	Mid	0.423	1.000	0.1388
		High	Low	0.003*	0.008*	**0.2083**
		Mid	Low	0.016*	0.047*	**0.0613**
	User view	High	Mid	0.804	1.000	0.1591
		High	Low	0.003*	0.008*	**0.229**
		Mid	Low	0.003*	0.009*	**0.0045**
	Total interaction	High	Mid	0.394	1.000	0.1718
		High	Low	0.001*	0.004*	**0.2042**
		Mid	Low	0.010*	0.029*	**0.0715**
Science	Assignment view	High High	Mid	0.617	1.000	0.2267
			Low	0.000*	0.001*	**0.2042**
		Mid	Low	0.000*	0.000*	**0.0035**
	Course content view	High High	Mid	0.396	1.000	0.115
			Low	0.021*	0.062	0.0897
		Mid	Low	0.002*	0.007*	**0.0233**
	User view	High High	Mid	0.345	1.000	0.0694
			Low	0.001*	0.003*	**0.1196**
		Mid	Low	0.009*	0.027*	**0.01**
	Total interaction	High High	Mid	0.568	1.000	0.1797
			Low	0.000*	0.001*	**0.1542**
		Mid	Low	0.002*	0.0058	0.0118

In the mathematics course, both high- and mid-achieving students interacted more with ‘Course Content View’, and ‘User View’, than low-achieving students, a result that is also observed for ‘Total Interactions’. Statistically significant comparisons between high- and low-achieving students in mathematics were observed to have medium to large effect sizes, whereas mid versus low-achieving students had small to medium effect sizes. Similar results were observed in the science course, where high-achieving students scored higher in specific interactivity measures (‘Assignment View’: η^2^ = 0.2042, ‘User View’: η^2^ = 0.1196, ‘Total Interactions’: η^2^ = 0.1542) than low-achieving students with medium to large effect sizes. There were no significant differences in interactivity measures between high- and mid-achieving students in mathematics or science.

### Research Question 2—Digital Literacy and Interactivity

**Fig. 4:**
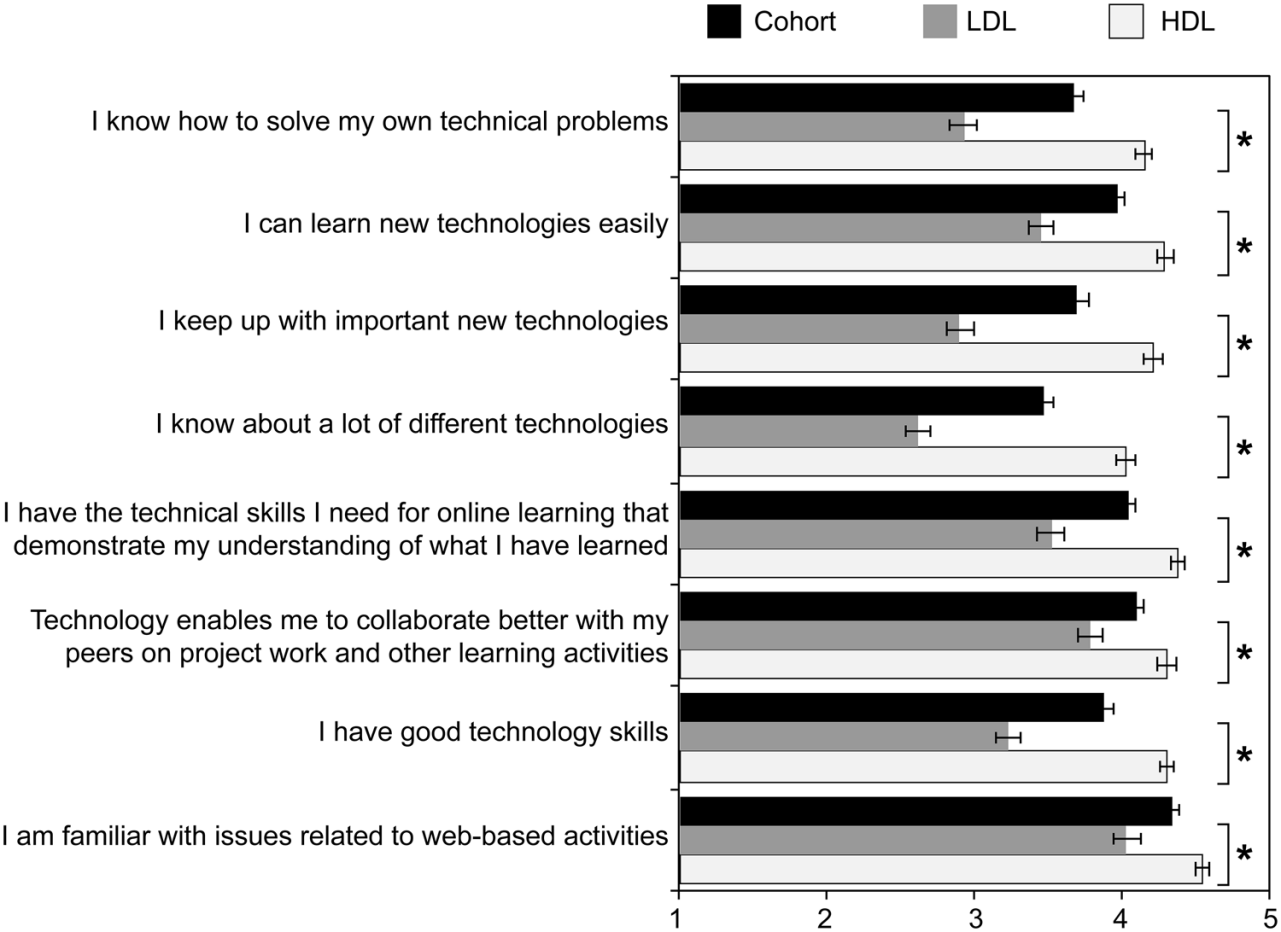
Average survey responses (1.0 = ‘Strongly Disagree’ to 5.0 = ‘Strongly Agree’) of high (HDL; *n* = 143)- and low (LDL; *n* = 91)-digital literacy students ± SEM for items in the ‘Digital Literacy’ scale

A student’s digital literacy has potential to impact on the extent to which they navigate virtual learning environments. In this study, a ‘digital literacy’ scale was adopted based on an adaptation of Ng’s digital literacy instrument (Ng, 2012), which includes three dimensions. The entire technical dimension cluster (6 items) was adapted to capture student perceptions of their technical capabilities in the current study context. This technical dimension of the instrument explores students’ ability to access digital resources for learning and retrieve information. The cognitive dimension and social-emotional dimensions of this instrument contained only two items in each cluster, and only one item from each dimension cluster was included in the current study. This decision was based on the small size of these clusters and their relevance to the research questions. Ng’s digital literacy instrument has become well characterised and retains reliability in different contexts even with adaptation of the number of items; for example, a recent study into the interplay between literacy and digital technology reported a Cronbach’s alpha of 0.91 for a total of 9 items and 249 participants (Nikou & Aavakare, 2021). A Cronbach’s alpha (*α*) analysis was conducted to assess the reliability of the responses from the adapted digital literacy scale (refer to Fig. 4 for items), which in this study produced a score of *α* = 0.847. The Cronbach’s alpha score was found to exceed 0.7, the minimum threshold for reliability (Fornell & Larcker, 1981). This indicated that the responses were internally consistent and of acceptable reliability.

**Fig. 5:**
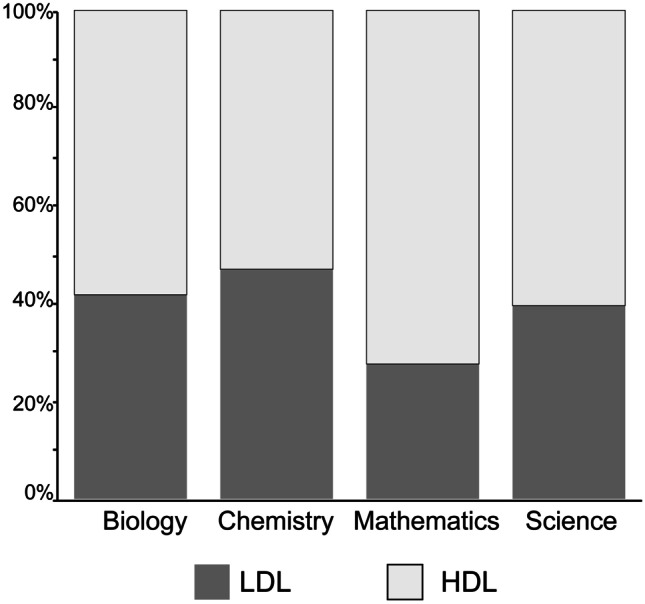
Distribution high (HDL; *n* = 143)- and low (LDL; *n* = 91)-digital literacy students

To group the students based on perceived digital literacy skills, a *k*-means cluster analysis was performed. The optimum number of clusters was *k* = 2; thus, a high-digital literacy (HDL; *n* = 143) and low-digital literacy (LDL; *n* = 91) group was formulated. The Mann–Whitney *U* test was conducted to observe the difference in HDL and LDL response to items in the ‘Digital Literacy’ scale. There is a significant difference between the two groups across all items, where the HDL mean (4.02–4.55 out of 5) is higher than the LDL mean (2.62–4.03 out of 5) (Fig. 4). This is to be expected as the groups were formulated based on their response to these items. In summary, two digital literacy groups, HDL and LDL, were able to be identified and there are both HDL and LDL students in each of the participating courses. The distribution of HDL and LDL students across the four courses can be seen in Fig. 5, and 52.89–72.13% were HDL and 27.87–47.11% were LDL students.

**Fig. 6:**
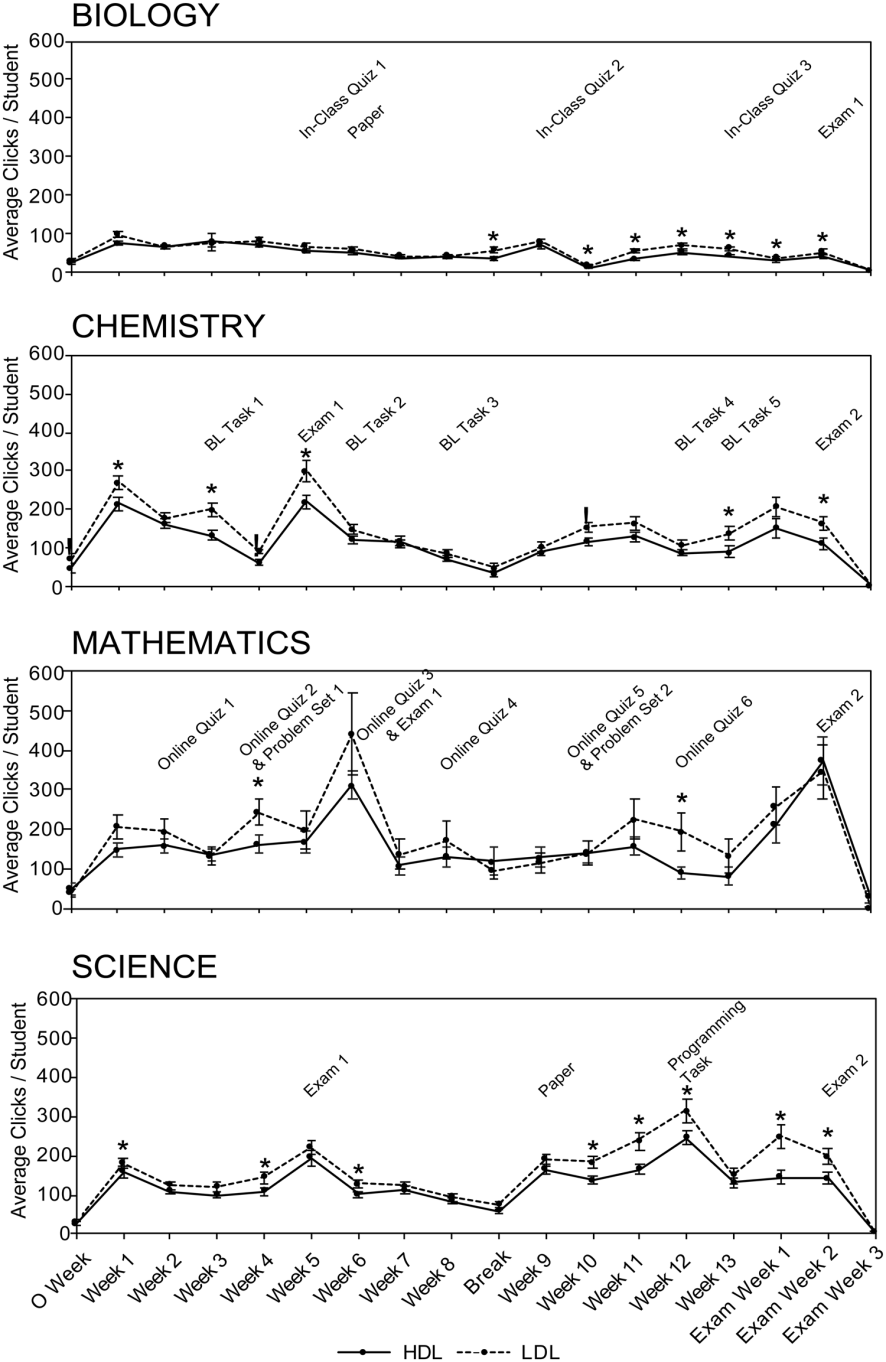
Comparison of average total clicks for high and low digital literacy students. Graphical representation of average total clicks per high (HDL)- and low (LDL)-digital literacy student ± SEM for each week of the semester. **A** Biology (*n* = 128). **B** Chemistry (*n* = 121). **C** Mathematics (*n* = 61), **D** Science (*n* = 117)

To examine the relationship between digital literacy and interactivity, the mean weekly ‘Total Interaction’ for HDL and LDL students was graphed (Fig. 6) and any assessment deadlines for each individual course have been indicated as well.

A Mann–Whitney *U* test was conducted for each week of the semester to determine if significant differences can be identified between the two digital literacy groups. The magnitude of interactivity in terms of click data (Fig. 6) is different in each course, which may be due to the relative differences in dependency on digital resources (Table 2). The level of navigational complexity in each course’s Blackboard page (Fig. 1) may be another contributing factor in the differences in interactivity across the courses. In addition, some courses provided supplementary content that was accessible in a parallel VLE (EdX Edge) used at the institution. Students need to go through a different number of folders to retrieve content, and the simplicity of biology’s VLE structure is corroborated by the range of its average clicks per week—2.45–96.46 clicks, less than what is seen in chemistry (3.05–299.6 clicks), mathematics (2.76–439.29 clicks), and science (3.52–314.5 clicks).

It is evident in Fig. 6 that at multiple points during the semester, LDL students complete significantly more interactions with the LMS than HDL students. While the majority of significantly different weeks occur around assessment deadlines, significant differences between digital literacy groups are also evident at times where there is no assessment. The amplitude of the disparity in clicks between LDL and HDL students is greater in chemistry and mathematics than in biology or science. Similar to the results based upon academic performance groups in these courses, the higher frequency of assessment in chemistry and mathematics may have led to a higher baseline level of online engagement for students in those courses. The overall lower number of clicks in biology in both LDL and HDL students can be attributed to the lack of LMS integration as it relates to the in-person paper-based in-class quizzes 1, 2, and 3.

When observing interactivity measures for digital literacy groups across the whole semester, ‘Total Interactions’ were observed to be significantly higher for the LDL group compared to the HDL group in three courses: biology (η^2^ = 0.0424), chemistry (η^2^ = 0.0768), and science (η^2^ = 0.0694). ‘Assignment View’ clicks were also observed to be significantly higher for the LDL group compared to the HDL group across the whole semester in the biology (η^2^ = 0.0761) and chemistry (η^2^ = 0.044). ‘Course Content’ clicks were also found to be significantly higher for the LDL group compared to the HDL group across the whole semester in the chemistry (η^2^ = 0.0687) and science (η^2^ = 0.0179) courses. The consistency of the observations across the courses appear to indicate that students in the LDL group interacted more with the LMS than students in the HDL group, especially in terms of interactions related to assessment and course content (Table 6).

**Table 6:** Descriptive statistics and non-parametric Mann–Whitney *U* statistical analysis of high (HDL)- and low (LDL)-digital literacy student students

**Course**	**Interactivity measure**	**HDL mean ± SEM**	**LDL mean ± SEM**	**Sig**	**η**^**2**^
Biology	Assignment view	61.7 ± 3.6	78.9 ± 4	0.000*	**0.0761**
	Course content view	455.3 ± 31.6	539.5 ± 39.5	0.059	0.0219
	Peer interaction	5.6 ± 1.7	4.2 ± 1.4	0.900	0.0031
	User view	48.8 ± 4.5	60.6 ± 6.7	0.170	0.0173
	Total interaction	797.1 ± 46.6	973.9 ± 59.6	0.022*	**0.0424**
Chemistry	Assignment view	114.6 ± 5.4	135 ± 6.8	0.009*	**0.044**
	Course content view	537.7 ± 41.1	723 ± 46.9	0.000*	**0.0687**
	Peer interaction	39.9 ± 6.8	37.7 ± 4.5	0.340	0.0006
	User view	57.7 ± 4.3	67.7 ± 5.3	0.089	0.0182
	Total interaction	1950.9 ± 127.3	2536.4 ± 134	0.000*	**0.0768**
Mathematics	Assignment view	96.6 ± 10.4	85.8 ± 8.6	0.772	0.0095
	Course content view	1635.9 ± 170.4	2027.5 ± 274.4	0.115	0.0291
	Peer interaction	12.8 ± 4.5	8.3 ± 4.3	0.966	0.0083
	User view	148.7 ± 23.1	155 ± 32.5	0.676	0.0005
	Total interaction	2706.9 ± 272.1	3273.7 ± 497.6	0.267	0.021
Science	Assignment view	178.2 ± 7.7	212.7 ± 12.7	0.072	0.0488
	Course content view	734.6 ± 59.8	848.5 ± 47.1	0.006*	**0.0179**
	Peer interaction	21.9 ± 6.7	17.7 ± 3.4	0.072	0.0024
	User view	65.8 ± 4.7	81 ± 6.9	0.068	0.0297
	Total interaction	2182.2 ± 119.5	2751.4 ± 158.6	0.003*	**0.0694**

After establishing a relationship between interactivity with the LMS, academic performance, and digital literacy, the effect of digital literacy on student academic performance was investigated. A Mann–Whitney *U* test was performed between HDL and LDL student’s academic performance as measured by the percentage weighting, on both overall grade and progressive assessment throughout the semester (Table 7). In biology, chemistry, and science courses, no significant differences in academic performance were observed between HDL and LDL students. Statistically significant differences were observed between HDL and LDL students in two assessment categories in the mathematics course—‘Online Quizzes’. Interestingly, it was LDL students who scored higher in these assessment items than HDL students, potentially a by-product of their increased interactivity with the LMS leading to improved performance. Similar trends were observed in biology, chemistry, and science, but increases in LDL student performance were not statistically significant when compared to HDL students in these courses. Moreover, high-achieving students were evenly divided between the HDL (51.35%) and LDL (48.65%) clusters across the 4 courses, so high-achieving students are not consistently the most digitally savvy within the cohort. It appears that overall LMS interactivity has more of an impact on academic performance than students’ self-perceived digital literacy in blended learning environments.

**Table 7:** Descriptive statistics and non-parametric Mann–Whitney *U* of high- (HDL) and low (LDL)-digital literacy students’ academic performance

**Course**	**Assessment (weighting in %)**	**HDL mean ± SEM %**	**LDL mean ± SEM %**	**Sig**	**η**^**2**^
Biology	Paper (7%)	6.1 ± 0.2	6 ± 0.2	0.054	0.0005
	Practical participation (3%)	2.7 ± 0.1	2.8 ± 0.1	0.159	0.0088
	In-class quizzes (40%)	28.7 ± 1.1	30 ± 1.1	0.599	0.005
	Exam 1 (50%)	29.2 ± 1.6	31.1 ± 1.6	0.519	0.0057
	Overall grade (100%)	66.7 ± 2.8	70 ± 2.8	0.614	0.0053
Chemistry	Lab reports (25%)	20.2±0.8	21 ± 0.8	0.144	0.0047
	BL tasks (5%)	4.3 ± 0.2	4.4 ± 0.2	0.397	0.0021
	Weekly online quizzes (10%)	7.9 ± 0.4	8.7 ± 0.3	0.062	0.0212
	Exam 1 (20%)	12.9 ± 0.6	14.2 ± 0.6	0.077	0.0212
	Exam 2 (40%)	20.8 ± 1.5	23.7 ± 1.5	0.142	0.0149
	Overall grade (100%)	66.1 ± 3.1	72.1 ± 3	0.119	0.0159
Mathematics	Problem sets (8%)	5.3 ± 0.4	6.2 ± 0.5	0.504	0.0316
	Online quizzes (8%)	4.5 ± 0.4	6.2 ± 0.5	0.013*	**0.1159**
	Practical participation (4%)	2.8 ± 0.2	3.4 ± 0.2	0.245	0.0542
	Computer exercise (10%)	7.8 ± 0.5	8.7 ± 0.6	0.145	0.026
	Exam 1 (20%)	8.2 ± 0.7	10.8 ± 1.1	0.047*	0.0705*
	Exam 2 (50%)	24.8 ± 2.3	31.8 ± 3.1	0.128	0.0571
	Overall grade (100%)	53.5 ± 4.2	67 ± 5.5	0.065	0.0661
Science*	Paper (15%)	9.8 ± 0.5	10.3 ± 0.5	0.625	0.006
	Programming task (15%)	9 ± 0.5	9.5 ± 0.6	0.556	0.0043
	Practical participation (100%)**	85.4 ± 3.4	94.2 ± 2.1	0.203	0.0365
	Exam 1 (10%)	59.3 ± 3.1	62.4 ± 3.3	0.586	0.0634
	Exam 2 (100%)***	5.7 ± 0.3	5.9 ± 0.3	0.871	0.0445
	Overall grade (100%)	63.3 ± 2.8	67.2 ± 2.9	0.456	0.0079

*Advanced science students had an extra Reflection Task (5%); however, this assessment has been excluded as advanced science students were inadequately represented

**Measured out of 100% as ‘Practical Participation’ was 10% for science students and 5% for advanced science students

*** ‘Exam 2’ weighting varied between 50 and 60% based on ‘Exam 1’ participation; thus, using their raw result as a percentage resolved this discrepancy

## Discussion

Successful use of online resources to acquire knowledge and demonstrate understanding has become a twenty-first century competency that learners need to be successful (Greene et al., 2018; Limniou et al., 2021). This study investigated factors that affect student interactivity with the LMS in four concurrently delivered BL STEM courses in the same semester in 2019. Two factors have emerged as important: the relationship between student digital literacy skills and interactivity, and that interactivity is correlated to academic performance. Interactivity is defined in this study as the relative measure of student interactions with content in the LMS captured as clickstream data. In this study, students’ digital literacy was related to academic achievement. The relationship between academic performance and interactivity in BL has been previously explored with inconclusive results (Ma & Lee, 2021). The role of digital literacy in student engagement across concurrent STEM courses has not been reported previously. Key findings emerging from our study can be categorised in terms of relationships between interactivity, digital literacy, and academic achievement. Additional nuances regarding differences in STEM discipline biases based on interactivity were also observed.

### Research Question 1—Academic Performance and Interactivity

When considering academic performance, high-achieving students interacted more with the LMS than low-achieving students. Weekly analysis of student LMS interactions revealed a difference between student performance groups across all times of the semester with respect to accessing course materials and assignments. This observation aligns with previous findings, where failing students were associated with relatively low activity or inactivity on VLE (Cohen, 2017). Several studies also found that assessment-related interactions were associated with higher academic performance (Kotsiantis et al., 2013; Mogus et al., 2012; Soffer & Cohen, 2019). Soffer and Cohen (2019) concluded that a willingness to complete assessment resulted in academic success. The greatest disparity in LMS interactivity between high- and low-achieving students was observed in chemistry and mathematics, courses with the highest number of progressive assessment items throughout the semester. Indeed, these findings may be amplified within specific disciplinary contexts; for example, increased student anxieties around perceived ease of use, quantitative skills, and technological capabilities have been observed in online chemistry courses (Faulconer & Griffith, 2021).

‘Peer Interactions’ were one of the lowest average number of clicks per student amongst the interactivity measures, a trend that has been reported in previous studies (Kotsiantis et al., 2013; Mogus et al., 2012; Soffer & Cohen, 2019). Soffer and Cohen (2019) hypothesised that since peer-collaboration through LMS is often not obligatory, students are less incentivised overall to participate in these interactions. In this study, significant differences were only observed when comparing high- and low-achieving students in the chemistry course, where the peer-related interactions were a part of the assessment: ‘BL Tasks’. Thus, this could be seen as a form of ‘Assessment View’ and reinforcing the importance of viewing assessment-related content. Kotsiantis et al. (2013) also recommended that communication with other students through LMS should be promoted by instructors to bolster engagement in a course’s online learning platform.

### Research Question 2—Digital Literacy and Interactivity

Students’ digital literacy skills were explored through their self-reported perceptions of their competencies, collected through an online survey using items sourced from a published instrument (Ng, 2012). By analysing their responses, two digital literacy groups were formulated, a high- (HDL) and low (LDL)-digital literacy group, through a *k*-means clustering analysis. HDL students on average responded higher to items in the ‘Digital Literacy’ scale than those in the LDL group, meaning that they were more confident in their technical capabilities.

The differences between HDL and LDL students’ ‘Total Interaction’ with LMS on a weekly basis were examined. In the weeks leading up to an exam, LDL students may be accessing course resources more than HDL students to study. These findings are similar to those previously reported, where students often completed assessment moments before the deadline (Kadoić & Oreški, 2018). The authors found that this applied to assessment that required revision across various course resources (e.g. past exam questions, solutions, and lecture notes). This pattern of activity may not apply to other assessment items in which students only need to access the LMS to submit the assessment (e.g. papers, online quizzes).

When exploring student interactions across the whole semester, LDL students were observed to interact more with the LMS than HDL students in ‘Assignment View’, ‘Course Content View’, and ‘Total Interactions’. This implies that LDL students may be less efficient with their LMS interactions, potentially clicking to a greater extent to find resources and achieve what HDL students might achieve in fewer clicks. Student perceptions of their digital literacy skills and their interactivity with a LMS to support this hypothesis; there is evidence that non-academic outcomes are important for BL hence indirectly impact on academic performance (Anthonysamy et al., 2020). In their review of studies that explored self-regulated learning skills in BL higher education environments, these authors identified that self-regulated learning strategies, cognitive engagement, motivational beliefs, and resource management influence academic success. It is an encouraging finding in the present study that LDL students were not observed to be disadvantaged in their overall academic achievement. Their increased LMS interactivity, which can be attributed to their learning approaches, positively correlates with academic performance. In fact, LDL students scored higher in online assessment tasks in mathematics than their HDL student counterparts which may indicate that this increased interactivity relates to the type of assessment. Further research is needed to explore this.

The transition from face-to-face learning activities into online environments and their impact on academic performance has been explored in a number of studies (Kemp & Grieve, 2014; Vo et al., 2017); there continues to be conflicting evidence in regard to whether overall academic performance is positively or negatively impacted when comparing face to face delivery to online learning. In this study, we have further explored the role of digital literacy in student engagement and academic performance to contribute to the growing body of evidence in the literature. Students with lower perceived digital literacy skills and high achievement interacted with LMS more than students with higher perceived digital literacy and low achievement in STEM blended courses. However, further research is required before it can be concluded that these two groups overlap.

## Significance of Findings and Recommendations for Practice in STEM BL Courses

This current study was completed in 2019, immediately prior to the impact of the COVID-19 pandemic and the need to shift into ERT. As we emerge into a post-COVID teaching paradigm, many academics will retain online teaching and assessment integrated with face-to-face activities meaning that BL will likely become the ‘new normal’ (Ma & Lee, 2021). Several insights are gained from this study that can inform future instructional design for BL courses moving forward after the ERT.

While reference to ‘STEM courses’ tends to aggregate a wide range of related disciplines, differences in activities and assessment should be acknowledged. In this study, we considered biology, chemistry, mathematics, and ‘science’ courses (the latter had a strong quantitative focus as students engaged with theory and practice in science). Laboratory learning was a face-to-face activity for biology and chemistry contexts in 2019 hence the LMS interactivity involved accessing instructional resources and related assessment. In 2020, these courses were forced to pivot into online virtual laboratories as part of the ERT response, a mode that had previously been recognised as promoting positive engagement due to novelty (Reeves & Crippen, 2021). There is no doubt that student interactivity with the LMS consequently is likely to have increased dramatically during 2020 and 2021; measures of student learning outcomes in virtual compared to face-to-face laboratory activities are yet to emerge. There are positive indicators that students possessed sufficient digital capabilities to succeed during ERT (Limniou et al., 2021) reinforcing the findings of Greene et al. (2018) who proposed that science students’ epistemic aims influenced their approaches to learning in tasks designed to promote understanding rather than knowledge acquisition.

We observed higher interactivity in chemistry and maths courses which appeared to be associated with assessment types. Chemistry anxiety and math anxiety are well established and can be further exacerbated when learning online, introducing multiple sources of anxiety (Faulconer & Griffith, 2021). Chemistry and math instructors might consider strategies for reducing anxiety related to online assessment tasks by introducing scaffolding to assist the navigation of the LMS and mitigating the effects of computer anxiety as a first step.

A recommendation of this study is that greater emphasis should be placed upon supporting students’ awareness and development of their digital literacy skills and competences to enhance academic success as well as non-academic skill development (including self-regulated learning, motivational beliefs, cognitive engagement, and resource management) in online or blended courses (Ng, 2012; Tang & Chaw, 2016). It is also recommended that instructors make strategies for accessing the LMS explicit, particularly where assessment is involved, and provide guidance for students at the beginning of a course in the form of a scaffolded online LMS orientation. This orientation can include productive learning sequences to access and interact with the online material, which clarifies instructor expectations for online learners (Buck, 2016). An orientation can either be formulated as an introductory module as part of students’ first year of online study at an institution, delivered as an extended orientation course that includes netiquette, time management, and self-regulated study skills for academic success (Korstange et al., 2020). The need for students’ digital upskilling should be balanced by well-structured online learning environments involving intuitive navigational prompts. As the perceived ease of use of online learning platforms has been positively correlated to students’ perception of timely graduation (Blau et al., 2016), online orientations can directly impact student retention across institutions with an expanding portfolio of online and blended courses during the COVID-19 pandemic.

The present study integrated quantitative data through two sources, and our findings can be enriched through qualitative analyses of student perceptions. Kotsiantis et al. (2013) argue that student perception data should be included in studies that adopt learning analytics. We acknowledge the limitation of the reliance on students’ self-reported data in the digital literacy scale for our study in the absence of a control group, which may impact on the generalisability of findings. However, insights gained in this study may contribute to aggregated findings in combination with other studies. We found through perception data combined with interactivity data that LDL students adopted a different approach to navigating the LMS in this study. Qualitative data can reveal greater insights into students’ engagement with digital literacy processes; for example, students’ epistemic cognition in science related to an assessment task has been explored through interviews (Greene et al., 2018). These researchers found evidence of different approaches to understanding versus acquisition based on students’ epistemic aims which aligns with the notion that BL environments promote non-academic outcomes (Anthonysamy et al., 2020). Epistemological beliefs, self-regulation, and digital literacy have also been found to be closely related for pre-service science teachers through a mixed methods study (Demirbag & Bahcivan, 2021). In the next phase of this research, a more granular exploration of student engagement with learning will be explored further in relation to their DL and beliefs through a mixed methods approach following the latter example.

## Data Availability

Raw data available on request.
